# Chromosomal Distribution of Ankylosing Spondylitis Susceptibility Loci

**DOI:** 10.31138/mjr.34.2.159

**Published:** 2023-06-30

**Authors:** Mostafa Saadat

**Affiliations:** Department of Biology, College of Sciences, Shiraz University, Shiraz, Iran

**Keywords:** ankylosing spondylitis, chromosome, genes, susceptibility

## Abstract

**Objectives:**

Previous studies have been indicated that susceptible loci of several multifactorial diseases were non-randomly distributed on human genome. There is no published data on chromosomal distribution of genes associated with risk of ankylosing spondylitis. Therefore, the present study was carried out.

**Methods:**

Published meta-analyses indexed in the PubMed database were used in the present study. Non-randomness chromosomal distribution of these loci was evaluated by the statistical method of Tai et al.

**Results:**

A total of 88 articles were obtained. There was 32 ankylosing spondylitis associated genes. The present study revealed that the human chromosome segments 6p11.2-p21.33, 19q13.2-q13.42, and 2q11.2-q14.1 were ankylosing spondylitis associated-rich regions by bearing 7, 6 and 4 susceptible loci, respectively.

**Conclusion:**

Ankylosing spondylitis associated-genes non-randomly have been distributed non-randomly on human chromosomes.

## INTRODUCTION

Although ankylosing spondylitis is one of the commonest rheumatic diseases worldwide, its pathogenesis is still unknown.^1,2^ Based on family and twin studies, it has significant heritability.^3^ In the early 1970s, studies identified a strong association between genetic polymorphism of HLA-B27 and the risk of ankylosing spondylitis, after that, many association studies have been examined the relationship between polymorphisms of candidate genes and the susceptibility to the disease.^4–28^ In post genome era, genome-wide association studies have been performed and a set consisting of several dozen of potential susceptible genetic variations have been indentified.^1,2,29^

It is well established that the human genome has a non-random structure at the chromosomal level.^30–36^ In the last few years, the chromosomal distributions of genes involved in several complex multifactorial diseases (such as breast and gastric cancers, schizophrenia and Parkinson disease) in humans have been studied.^36–42^ Considering that the distribution of ankylosing spondylitis associated genes has not been reported, the current study was carried out.

## METHODS

Published meta-analyses indexed in the PubMed database were used in the present study. Keywords for searching were ankylosing spondylitis, meta-analysis and polymorphism. No time limitation was considered for the results and for selection of the studies. Last update was 27 August 2022. Meta-analysis study design was eligible for including in the study. List of the references of the relevant articles, as well as data presented in two articles (Pedersen et al., and International Genetics of Ankylosing Spondylitis Consortium [IGAS])^2,29^ were also searched to exclude the possibility of missing any eligible study. A total of 88 articles were obtained. There were 11 studies with other purposes, such as those examine association of ankylosing spondylitis and cancers which were excluded. After removing these articles, the author screened the retrieved studies. For some genetic polymorphisms which more than one meta-analyses were published about their association with the risk of ankylosing spondylitis, the latest articles were included in the study and other articles were excluded. Loci reported from genome-wide association studies were not included in the analysis.

Genes with at least one genetic variation associated with the susceptibility to ankylosing spondylitis were included in the present study.^4–28^ Statistical relationship between polymorphisms and the risk of the disease may had been stated for at least one human ethnicity.

A non-electronic hand-written form was used for data extraction. Data regarding article identification (authors name and publication date) as well as main study attributes (gene name and locus symbol) were extracted from each selected study, by the author. Cytogenetic location and MIM (Mendelian inheritance in Man ID) of the susceptible loci were also extracted from Home - OMIM database (https://www.omim.org).

To examine the non-randomness chromosomal distribution of ankylosing spondylitis associated genes, the statistical method of Tai et al., (1993) was used.^43^ The relative width of each band was measured using the International System for Chromosome Nomenclature based on 400 bands. In inferential statistics, an important kind of statistical error is the mistaken rejection of a null hypothesis as the result of a statistical comparison. This is a false positive result and is called type I error. Considering that a small number of ankylosing spondylitis susceptible loci were included in the present study, in order to reduce the statistical type I error, a P-value less than 0.001 was considered statistically significant.

## RESULTS AND DISCUSSION

After removing 11 unrelated studies and older articles concerning association a specific polymorphism with the risk of ankylosing spondylitis, as described in Methods section, 25 articles were included in the study.^4–28^ A total 32 of susceptible loci were identified. Extracted data (full gene name, locus symbol, MIM, and gene cytogenetic location) were summarized in **Table 1**.

**Table 1.** Chromosomal locations of genes associated with the risk of ankylosing spondylitis.

**Genes**	**Gene symbols**	**MIM**	**Cytogenetic locations**	**Ref.**
Protein tyrosine phosphatase, nonreceptor-type, 22	*PTPN22*	600716	1p13.2	20
Interleukin 23 receptor	*IL23R*	607562	1p31.3	25
Homocystinuria due to deficiency of n (5,10)-methylenetetrahydrofolate reductase activity	*MTHFR*	236250	1p36.22	14
Interleukin 10	*IL10*	124092	1q32.1	21
2p15	*2p15*		2p15	28
Interleukin 1 receptor, type i	*IL1R1*	147810	2q11.2-q12.1	8
Interleukin 1-alpha	*IL1A*	147760	2q14.1	6
Interleukin 37	*IL37*	605510	2q14.1	6
Interleukin 1 receptor antagonist	*IL1RN*	147679	2q14.1	9
Cytotoxic t lymphocyte-associated 4	*CTLA4*	123890	2q33.2	24
Programmed cell death 1	*PDCD1*	600244	2q37.3	5
Anthrax toxin receptor 2	*ANTXR2*	608041	4q21.21	23
Nuclear factor kappa-b, subunit 1	*NFKB1*	164011	4q24	26
Endoplasmic reticulum aminopeptidase 1	*ERAP1*	606832	5q15	4
Micro RNA 146a	*MIR146A*	610566	5q33.3	22
Interleukin 17f	*IL17F*	606496	6p12.2	19
Interleukin 17a	*IL17A*	603149	6p12.2	19
Proteasome subunit, beta-type, 9	*PSMB9*	177045	6p21.32	16
Transporter, ATP-binding cassette, major histocompatibility complex, 1	*TAP1*	170260	6p21.32	17
Transporter, ATP-binding cassette, major histocompatibility complex, 2	*TAP2*	170261	6p21.32	17
Tumor necrosis factor	*TNF*	191160	6p21.33	15
Major histocompatibility complex, class i, b	*HLA-B*	142830	6p21.33	12
Tumor necrosis factor receptor superfamily, member 1A	*TNFRSF1A*	191190	12p13.31	10
Vitamin D receptor	*VDR*	601769	12q13.11	27
Signal transducer and activator of transcription 3	*STAT3*	102582	17q21.2	7
Transforming growth factor, beta-1	*TGFB1*	190180	19q13.2	13
Killer cell immunoglobulin-like receptor, two domains, short cytoplasmic tail, 1	*KIR2DS1*	604952	19q13.4	18
Killer cell immunoglobulin-like receptor, two domains, short cytoplasmic tail, 5	*KIR2DS5*	604956	19q13.4	18
Killer cell immunoglobulin-like receptor, two domains, long cytoplasmic tail, 2	*KIR2DL2*	604937	19q13.4	18
Killer cell immunoglobulin-like receptor, two domains, short cytoplasmic tail, 2	*KIR2DS2*	604953	19q13.4	18
Killer cell immunoglobulin-like receptor, three domains, long cytoplasmic tail, 1	*KIR3DL1*	604946	19q13.42	18
Cytochrome p450, subfamily IID, polypeptide 6	*CYP2D6*	124030	22q13.2	11

**Figure 1.**
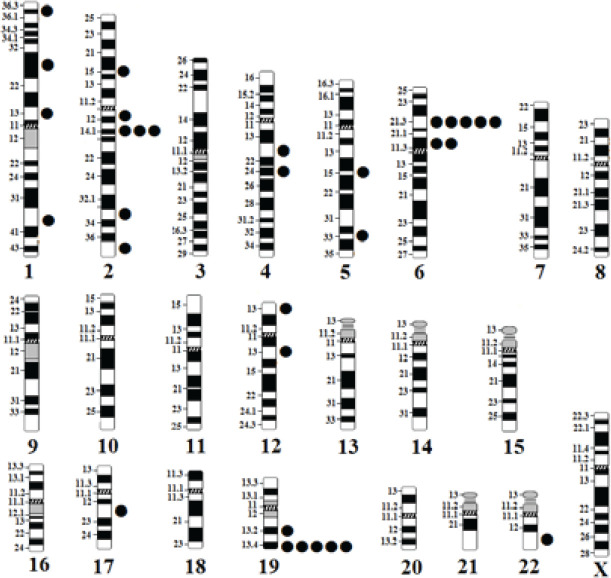
Ankylosing spondylitis associated genes distribution on chromosomes.

From 32 ankylosing spondylitis associated genes, 7 (*IL17F*, *IL17A*, *PSMB9*, *TAP1*, *TAP2*, *TNF*, and *HLA-B*), 6 (*TGFB1*, *KIR2DS1*, *KIR2DS5*, *KIR2DL2*, *KIR2DS2*, and *KIR3DL1*) and 4 (*IL1R1*, *IL1A*, *IL37*, and *IL1RN*) genes were located on the human 6p11.2-p21.33, 19q13.2-q13.42, and 2q11.2-q14.1 chromosome segments, respectively. This finding supports the non-randomness distribution of diseases associated genes on human chromosomes.^37–42^ There was no statistical evidence that the other ankylosing spondylitis associated-genes distributed non-randomly on the chromosomes (**Figure 1**).

One of the important featured of multifactorial complex traits such as ankylosing spondylitis is that different polymorphic genes/alleles are involved in the pathogenesis. Each of above-mentioned chromosomal segments bearing several functional loci. We know that these loci are in linkage disequilibrium and their allelic frequencies differ between ethnic groups. Additionally, additive, dominance and epistatic effects might be involved between the susceptible genes. There is evidence that some of known polymorphic alleles involved in the pathogenesis of ankylosing spondylitis have synergistic and epistatic effects with each other.^44–46^

Application of knowledge gained from nonrandom accumulation of ankylosing spondylitis associated loci on the human chromosome segments 6p11.2-p21.33, 19q13.2-q13.42, and 2q11.2-q14.1 could be of crucial importance in development of a laboratory diagnostic test for mass screening programs to find high risk persons.

## Data Availability

All data are included in **Table 1**.
